# MHC-I and PirB Upregulation in the Central and Peripheral Nervous System following Sciatic Nerve Injury

**DOI:** 10.1371/journal.pone.0161463

**Published:** 2016-08-23

**Authors:** André Luis Bombeiro, Rodolfo Thomé, Sérgio Luiz Oliveira Nunes, Bárbara Monteiro Moreira, Liana Verinaud, Alexandre Leite Rodrigues de Oliveira

**Affiliations:** Department of Structural and Functional Biology, Institute of Biology, University of Campinas – UNICAMP, Rua Monteiro Lobato, 255, CEP: 13083–865, Campinas, SP, Brazil; Stony Brook University, UNITED STATES

## Abstract

Major histocompatibility complex class one (MHC-I) antigen-presenting molecules participate in central nervous system (CNS) synaptic plasticity, as does the paired immunoglobulin-like receptor B (PirB), an MHC-I ligand that can inhibit immune-cells and bind to myelin axon growth inhibitors. Based on the dual roles of both molecules in the immune and nervous systems, we evaluated their expression in the central and peripheral nervous system (PNS) following sciatic nerve injury in mice. Increased PirB and MHC-I protein and gene expression is present in the spinal cord one week after nerve transection, PirB being mostly expressed in the neuropile region. In the crushed nerve, MHC-I protein levels increased 2 weeks after lesion (wal) and progressively decreased over the next eight weeks. The same kinetics were observed for infiltrating cytotoxic T lymphocytes (CTLs) but not for PirB expression, which continuously increased. Both MHC-I and PirB were found in macrophages and Schwann cells but rarely in axons. Interestingly, at 8 wal, PirB was mainly restricted to the myelin sheath. Our findings reinforce the participation of MHC-I and PirB in CNS plasticity events. In contrast, opposing expression levels of these molecules were found in the PNS, so that MHC-I and PirB seem to be mostly implicated in antigen presentation to CTLs and axon myelination, respectively.

## Introduction

The major histocompatibility complex (MHC) is encoded by a polymorphic gene *locus* responsible for the transduction of antigen-presenting molecules, such as the human leucocyte antigen (HLA) and the histocompatibility molecules in mice (H). MHC class one molecules are expressed by nucleated cells and are recognized by cytotoxic T lymphocytes (CTLs or CD8 T cells), which can be activated in the presence of non-self-peptides, triggering the elimination of the target cell. MHC-I molecules are composed by one heavy transmembrane alpha-chain coupled with a β-2 microglobulin (β-2m) subunit. The antigen, derived from cytosolic proteins, is allocated in a cleft formed in the alpha subunit and is important for the stability of the complex on the cell membrane [1].

For many years, it was believed that neurons did not express significant amounts of MHC-I, which allowed them to be immune to CTL recognition. This provided the basis for the concept of the immune-privileged status conferred to the central nervous system (CNS). However, it has been demonstrated that the expression of MHC-I molecules and components involved with the MHC-I signaling pathway could be upregulated by the administration of interferon (IFN)-γ in electrically silenced hippocampal neurons *in vitro* [2, 3]. Moreover, previous studies have shown that MHC-I molecules are expressed in the CNS during different phases of development [4] and aging [5] as well as after injuries [6, 7]. In this sense, Oliveira and colleagues [8] reported the importance of MHC-I expression for the maintenance of inhibitory synapses opposed to axotomized alpha-motorneurons. In that study, the authors showed higher synapse detachment in the absence of MHC-I following peripheral axotomy, with a decreased presence of inhibitory inputs and impaired axonal regeneration [8]. Subsequent studies corroborated the involvement of MHC-I molecules in synaptic plasticity. In this sense, the upregulation of MHC-I induced by IFN-β interfered with the synaptic reactivity and increased astroglial response in the spinal cord [7], as well as improved axonal growth and motor function recovery [6], following peripheral nervous system (PNS) damage. Moreover, diminished MHC-I expression and reduced astrogliosis correlated with decreased synaptic stripping in C57BL/6 mice one week after axotomy [9]. Nevertheless, to date, the precise role of MHC-I in CNS/PNS plasticity is not fully understood.

The paired immunoglobulin-like receptor B (PirB) is an MHC-I ligand and is expressed by several hematopoietic cells, including macrophages, dendritic cells, B cells, mast cells, and granulocytes [10]. PirB is composed of six extracellular immunoglobulin-like domains, a transmembrane segment, and a cytoplasmic tail containing four intracellular immunoreceptor tyrosine-based inhibition motifs (ITIMs). The phosphorylation of ITIMs by Src family kinases results in the recruitment of phosphatases, such as Src homology 2-containing protein tyrosine phosphatase (SHP)-1 and SHP-2, unleashing downstream cell inhibitory responses [10]. In neutrophils, PirB is involved in the reduction of the oxidative burst, secondary granule release, and hyperadhesiveness. In macrophages, PirB was shown to negatively regulate adhesiveness and cell spreading [11]. In addition to MHC-I molecules, PirB also binds to myelin inhibitors of axon regeneration, such as Nogo, MAG, and OMgp, and interferes with neuronal plasticity [12]. Recently, PirB has been reported to be a receptor for β-amyloid proteins, mediating the loss of synaptic plasticity in a mouse model of Alzheimer’s disease [13]. Furthermore, PirB was also shown to be important for restricting ocular dominance plasticity in an MHC-I-dependent manner [14]. In the PNS, the role of PirB remains poorly understood. Recently, Thams and colleagues [15] reported a subpopulation of Schwann cells expressing PirB after sciatic nerve transection and suggested that these cells interact with damaged motor neurons via MHC-I molecules, inhibiting axon growth.

In view of the dual role developed by MHC-I and PirB in the immune and nervous systems, together with their involvement in cellular plasticity, the present study investigated the expression and distribution of such molecules in the PNS and CNS under basal conditions and following injury. We found both MHC-I and PirB to be upregulated in the CNS one week after peripheral nerve injury, when plasticity events are taking place, whereas in the PNS, opposing expression levels where depicted during the regenerative process.

## Materials and Methods

### Animals

Male C57BL/6 mice (6–8 weeks-old) were obtained from the Multidisciplinary Center for Biological Investigation of the State University of Campinas (CEMIB/UNICAMP) and kept in our animal facility in cages/ventilated racks under a 12 h light/dark cycle, with controlled temperature (21–23°C) and humidity (30%) and free access to water and food. All experiments concerning animal handling were approved by the Institutional Committee for Ethics in Animal Experimentation (Committee for Ethics in Animal Use—Institute of Biology—CEUA/IB/UNICAMP, protocol number 2524–1) and were performed in accordance to the guidelines of the Brazilian College for Animal Experimentation.

### Experimental Procedures

#### Sciatic nerve crushing

Ketamine (Fort Dodge, USA, 100 mg/Kg) and xilasin (König, Argentina, 10 mg/Kg) anesthetized mice were subjected to left sciatic nerve crushing at the sciatic notch level with a non-serrated forceps (number 4), by applying a constant pressure for 30 s. At 2, 4, and 8 weeks after lesion (wal), mice (n = 6 in each time-point) were deeply anesthetized (ketamine 400 mg/Kg, xilasin 40 mg/kg) and euthanized by transcardial perfusion using ice cooled phosphate buffered saline (PBS 0.1 M, pH 7.4) followed by cold fixative solution (formaldehyde 10% in PB 0.1 M, pH 7.4) for histological procedures. For flow cytometry analysis, animals were not perfused with fixative, and the crushed nerves were dissected out.

#### Sciatic nerve transection

Mice were anesthetized as above and submitted to the transection of the left sciatic nerve at the obturator tendon level. A 2 mm gap was left to prevent nerve regeneration. 1 wal, mice were euthanized as previously described, and the spinal cord was dissected (from L4 to L6) for histological (n = 6) or real time PCR (n = 6) procedures. In the last case, mice were not perfused with formaldehyde, and the ipsilateral side of the lumbar spinal cord was immediately dissected in an ice-cold sterile petri dish and frozen in liquid nitrogen. Non-operated mice (n = 6) were used as a negative control and were submitted to the same experimental procedures.

### Tissue preparation and sampling for immunofluorescence

Sciatic nerve and lumbar spinal cord samples were post-fixed in paraformaldehyde 4% (overnight, 4°C), washed in PBS, and stored in sucrose 10% and 20% (w/v, overnight each concentration, 4°C). Samples were embedded in tissue freezing medium (Tissue Tek, Sakura Finetek), frozen in liquid nitrogen-cooled N-hexane (-35°C), and sectioned (12 μm) in a cryostat (Microm, HM 525). Non-adjacent sciatic nerve sections were placed in gelatin-coated glass slides (one and every 11^th^ section, 2–3 sections per slide) and stored at -20°C until used. For the spinal cord, four sequential sections were mounted on each glass slide up to the end of the sample and kept at -20°C.

### Immunofluorescence

Samples were washed in PB 0.1 M, blocked with BSA 3% in PB 0.1 M (except for PirB; 1 h, room temperature), and incubated with the following primary antibodies (overnight, 4°C): rat anti-H-2^b^ (for MHC-I molecules; 1:100, BMA Biomedicals, Cat. code: T-2105, clone: ER-HR52), goat anti-Lilrb3 (for PirB, 1:200, Santa Cruz Biotechnologies, Cat. code: sc-9609), rabbit anti-S100 (1:1500, Dako, Cat. code: Z0311), rabbit anti-CD31 (1:100, Santa Cruz Biotechnologies, Cat. code: sc-8306) and rat anti-CD8a FITC labeled (1:100, eBioscience, Cat. code: 11–0081). For double immunolabeling using anti-PirB antibodies we employed one of the following primary antibodies: mouse anti-neurofilament (1:4000, Dako, Cat. code: M0762), rabbit anti-S100 (1:1500), rabbit anti-Iba1 (1:700, Wako, Cat. code: 019–19741), rabbit anti-GFAP (1:500, Abcam, Cat. code: AB7779), and rabbit anti-NeuN (1:500 or 2 μg/mL, Millipore, Cat. Code: ABN78). Sections were washed with PB 0.1 M and incubated with one or two of the following secondary antibodies (45 min, room temperature): Cy2-AffiniPure goat anti-mouse IgG (1:500, Jackson ImmuneResearch, Cat. code: 115-225-146), Cy3-AffiniPure donkey anti-mouse IgG (1:500, Jackson ImmuneResearch, Cat. code: 715-165-150), Cy2-AffiniPure donkey anti-rabbit IgG (1:500, Jackson ImmuneResearch, Cat. code: 711-225-152), Cy3-AffiniPure donkey anti-rabbit IgG (1:500, Jackson ImmuneResearch, Cat. code: 711-165-152), Cy3-AffiniPure donkey anti-rat IgG (1:500, Jackson ImmuneResearch, Cat. code: 712-165-150) and Alexa Fluor 546 donkey anti-goat IgG (1:700, Molecular Probes, Cat. code: A-11056). For the CD8a labeling, a secondary antibody was not necessary. Samples were rinsed and incubated with PB 0.1 M containing DAPI (1:10,000; 10 min). Coverslips were mounted with anti-fade media (ProLong^®^ Gold antifade reagent, Invitrogen, Cat. code: P36930). All antibodies, except anti-PirB, were diluted in a PB 0.1 M solution containing BSA 1% (w/v) and Triton X-100 0.2% (v/v). For PirB detection, samples were blocked with 5% donkey serum in PB 0.1 M (v/v; 1 h, room temperature), and the primary and secondary antibodies were diluted in PB 0.1 M containing 1.5% donkey serum (v/v) and 0.2% Triton X-100 (v/v). For H-2^b^ detection, samples were immersed in cooled acetone (-20°C, 1 min) for antigenic reconstitution before blocking. *In vitro* microglia immunolabeling (PirB/Iba1) was performed as above.

### Image acquisition and immunolabeling quantification

Representative images of each sample were acquired using a fluorescence microscope (Nikon Eclipse TS100 with digital camera Nikon DXM1200F or Leica DM5500B with digital camera Leica DFC345 FX) for immunolabeling quantification. For sciatic nerve samples, five random fields were chosen from two tissue sections immediately distal to the crush site and up to 5 mm from the lesion (S1A Fig). For the spinal cords, one field from each section was photographed in the ventral horn, in the area affected by the nerve transection (S1B Fig). Four sections per sample were obtained from the middle of the lumbar intumescenses. Pictures were analyzed with the ImageJ software (version 1.45s, National Institute of Health, USA), and the integrated density of pixels (IDP) was obtained by applying the enhance contrast and density slicing feature in a fixed area. Then, the IDP mean value of each sample was used, and data were expressed as the ratio of the operated groups to their respective controls (non-operated). For orthogonal projections (where indicated), images were acquired using a fluorescence microscope (Leica DM5500B with digital camera Leica DFC345 FX), and the z stack varied from 30 to 40 layers. The projections were done following 3D deconvolution (total interactions: 10; refractive index: 1.52), and both were performed using the Leica LAS AF software.

### Flow Cytometry

Dissected nerves were mechanically dissociated with a cell strainer (70 μm pores), and cells were collected in Petri dishes with cold PBS. To increase intracellular protein production, cells were treated for 3 h at 37°C with Phorbol 12-myristate 13-acetate (PMA, 50 ηg/mL, Sigma) and Ionomycin (500 ηg/mL). Brefeldin A (1 μg/mL) was added to prevent protein release by the cells. Samples were fixed and permeabilized with commercial buffers (Fixation/Permeabilization buffers, eBioscience, Cat. code: 00–5521) according to the manufacturer’s instructions, then washed with PBS, and incubated with antibodies as follows: anti-CD8/FITC, anti-IL-10/APC and anti-IFN-g/PE. 30,000 events were acquired from each sample in the flow cytometer (FACS Canto, BD Biosciences, Franklin Lakes, NJ, USA) and analyzed in FlowJo 10.0.5 software (Tree Star Inc., Ashland, OR, USA). Gating for flow cytometry was performed using unstained cells and FMO (Fluorescence Minus One) analysis, as described elsewhere [16]. For cell staining, we used these antibodies: anti-mouse CD8a FITC (0.5 μg, eBioscience, Cat. code: 11–0081), anti-mouse IFN-γ PE (0.5 μg, eBioscience, Cat. code: 12–7311) and anti-mouse IL-10 APC (0.5 μg, eBioscience, Cat. code: 17–7101).

### Real Time RT-PCR

Total RNA was isolated from the lumbar intumescence using a commercial kit (RNeasy Lipid Tissue Kit, Qiagen, Cat. code: 74804) and then quantified in a spectrophotometer (Nanophotometer, Implen). cDNA was obtained from 1 μg total RNA also using a commercial kit (Agilent Technologies, Affinity Script QPCR cDNA Synthesis Kit, Cat. code: 600559) and then amplified (1 μL sample) in a thermocycler (Stratagene Mx3005-P, Agilent Technologies) using SYBR Green reagent (Agilent Technologies, Brilliant II SYBR Green QPCR Master Mix, Cat. code: 600828) and the following primers (5 pmol): *Pirb* (F:GTCTGTGGCCTTCATCCTGTTCC, R:TGTTCAGCTCCACTCCATCCTCAG) [17], *B2m* (F:ATGGCTCGCTCGGTGACCCTG, R:CCGGTGGGTGGCGTGAGTATACTT) and *Gapdh* (F:TGCACCACCAACTGCTTA, R:GGATGCAGGGATGATGTTC). All procedures were performed according to the manufacturer’s instructions. Samples were analyzed in triplicate, and mRNA levels were obtained by the normalization of the target gene to the endogenous reference (G*apdh*) and then relativized to the calibrator samples (non-operated) using the formula 2^-ΔΔCt^.

### Statistical analyses

Sciatic nerve data analyses were performed by the one-way analysis of variance (ANOVA) followed by Bonferroni’s multiple comparison tests. For the spinal cord we employed the unpaired *t* test. Data are expressed as the mean ± standard error mean (SEM) and *p* values <0.05 were considered significant. All statistical analyses were performed using GraphPad Prism version 4.00, GraphPad Software, San Diego, CA, USA.

## Results

### Sciatic nerve crushing-induced MHC-I expression decreases over time

Although it is known that the damaged PNS may produce MHC-I molecules [6], we were interested in performing a kinetic evaluation of the MHC-I expression in the crushed sciatic nerve during the regenerative process. For that purpose, we monitored motor recovery by assessing the sciatic function index (S1 File and S2 Fig) and set our experimental groups at the following time points: when motor function was fully reestablished at 4 weeks after lesion (4 wal) and in half and twice the recovery time (2 and 8 wal, respectively).

**Fig 1:**
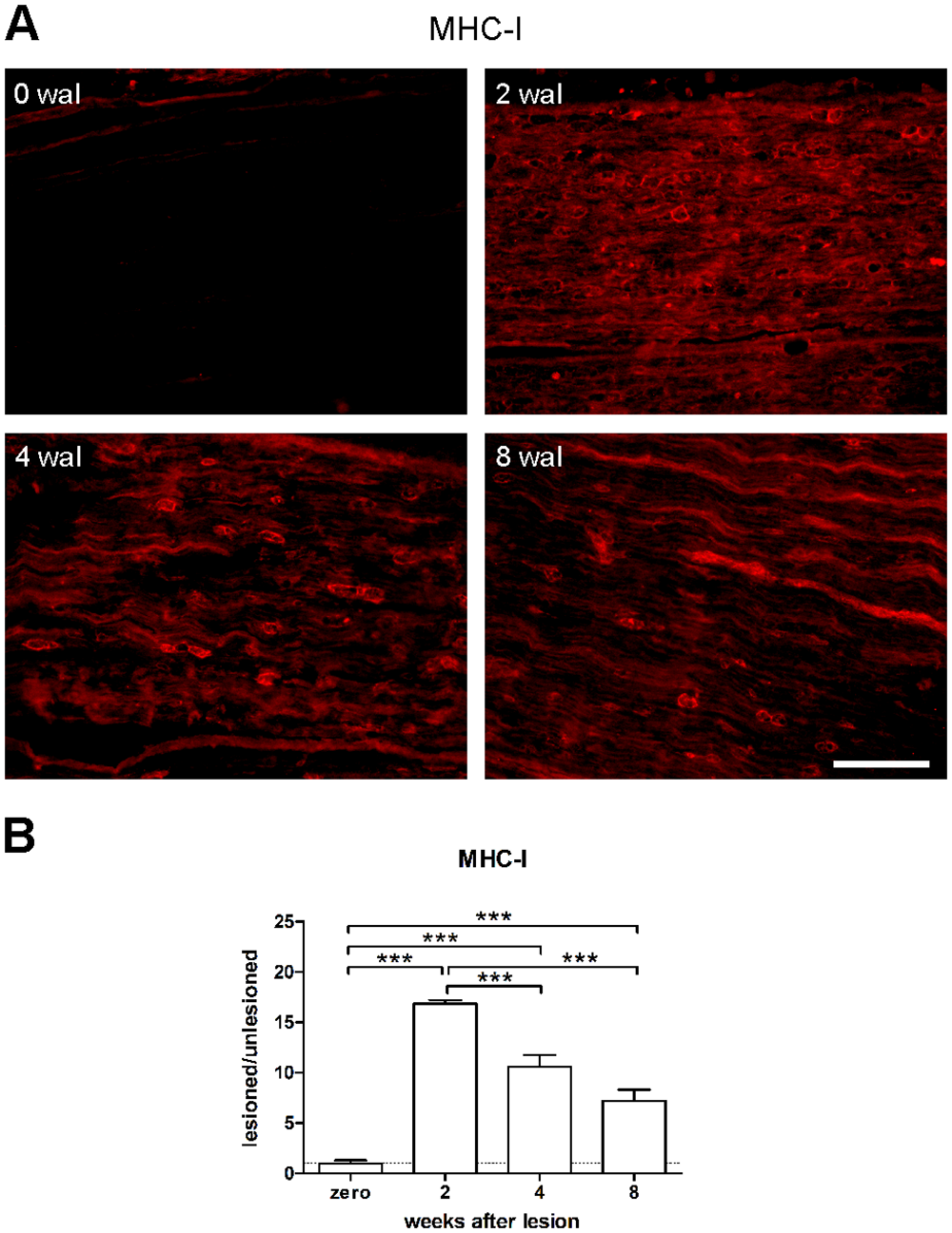
Increased expression of MHC-I molecules in the sciatic nerve after crushing. Mice were submitted to the crushing of the sciatic nerve. 2, 4, and 8 weeks after lesion (wal), nerves were dissected out and analyzed by immunofluorescence for MHC-I expression. Uninjured mice were used as a control (zero wal). A) representative images of MHC-I labeling at all time-points. Scale bar: 100 μm. B) MHC-I quantification by the integrated density of pixels method (lesioned/unlesioned). Data are presented as the mean ± SEM. n = 6 in each time point. ***p<0.001 according to the one-way ANOVA, followed by Bonferroni post-tests.

**Fig 2:**
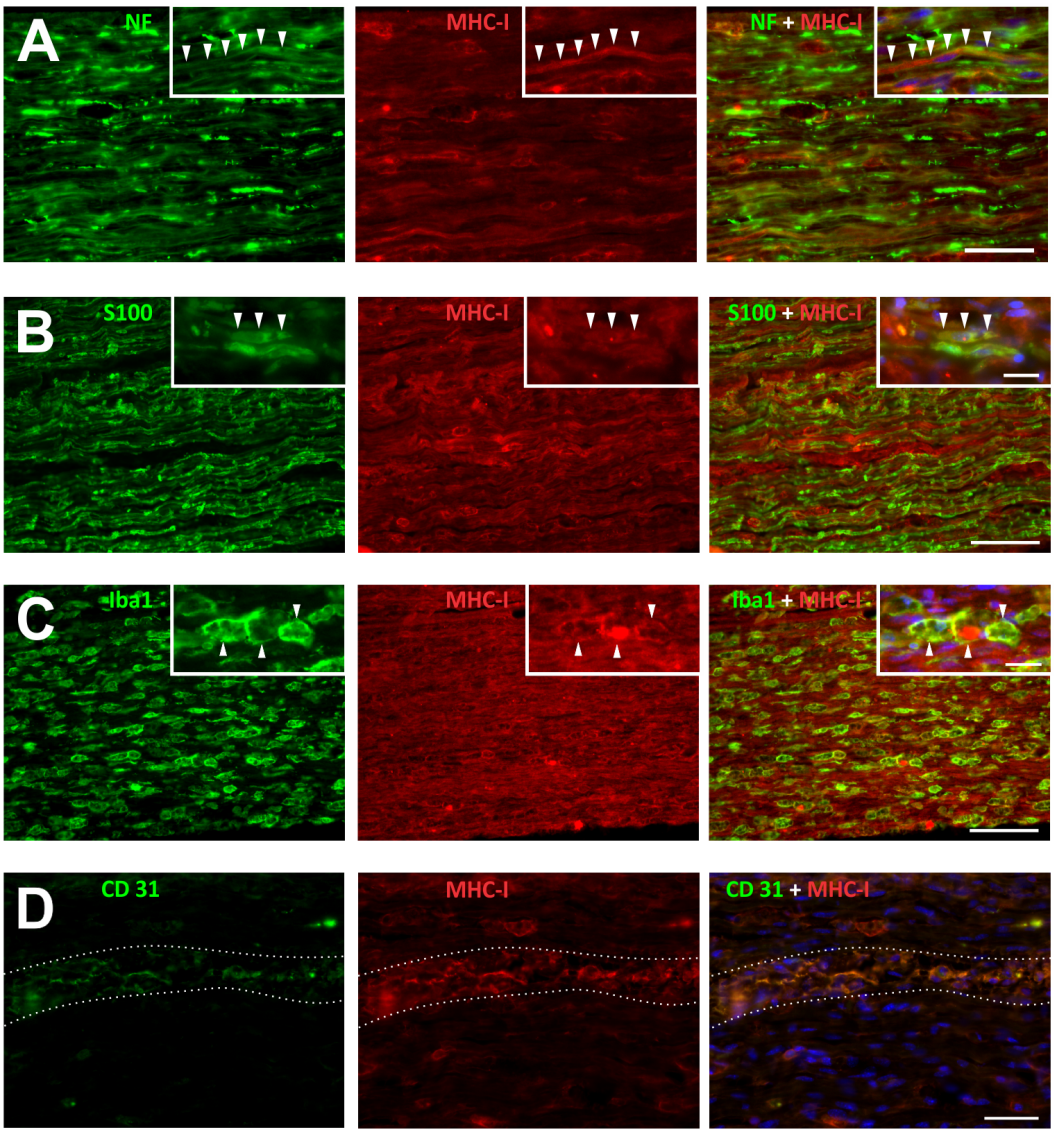
Sciatic nerve MHC-I expression by distinct cellular sources after crushing. Double immunolabeling of MHC-I molecules and A) neurofilament (NF, axonal marker), or B) S100 protein (Schwann cell marker) or C) Iba1 protein (macrophage marker) after crushing. A) Most of the axons expressed zero or low MHC-I immunolabeling. Inset: MHC-I^+^ axon (arrowheads). B) MHC-I expression by Schwann cells. Inset: MHC-I labeled Schwann cell (arrowheads). C) Macrophages expressing MHC-I after injury. In detail, MHC-I labeled macrophages (arrowheads), one of which with cytoplasmic MHC-I immunolabeling. D) MHC-I expression by endothelial cells. A blood vessel is highlighted (dashed lines). Nuclei are DAPI positive, NF: neurofilament, CD31: endothelial cell adhesion molecule. Time-points: A and D, 4 weeks after lesion (wal); B and C, 2 wal. Scale bars: A and D, 50 μm; B and C, 100 μm; insets, 20 μm.

According to the immunohistochemistry analysis, MHC-I expression peaked at 2 wal and decreased thereafter. However, the expression did not recover to baseline values at 8 wal (Fig 1A and 1B, S2 File). Double immunostaining analysis revealed that MHC-I expression is particularly low or absent in axons, both under basal conditions and after damage (Fig 2A), during all time-points. Fig 2A inset shows an MHC-I^+^ axon during the regenerative process. MHC-I labeled Schwann cells were not visualized in the undamaged nerve. However, MHC-I expression by these cells increased after injury, being more prominent at 2 wal (Fig 2B) and 4 wal. Macrophages also expressed MHC-I. However, the expression was more intense in the round-shape activated cells (Fig 2C). Accordingly, once inflammation was resolved, fewer MHC-I^+^ macrophages were observed. Moreover, MHC-I immunolabeling was weak or absent in ramified macrophages that could easily be observed in the regenerating tissue at 4 and 8 wal, as well as in the undamaged control. In Fig 2C, one MHC-I^+^ cell inclusion in a macrophage is shown. Importantly, MHC-I was also expressed by infiltrating immune-cells, epineural and perineural cells, and blood vessels. Accordingly, once regeneration occurred, the remaining MHC-I immunolabeling was mostly restricted to those structural elements, in particular the blood vessels (Fig 2D).

### CD8 T cell frequency decrease over time

**Fig 3:**
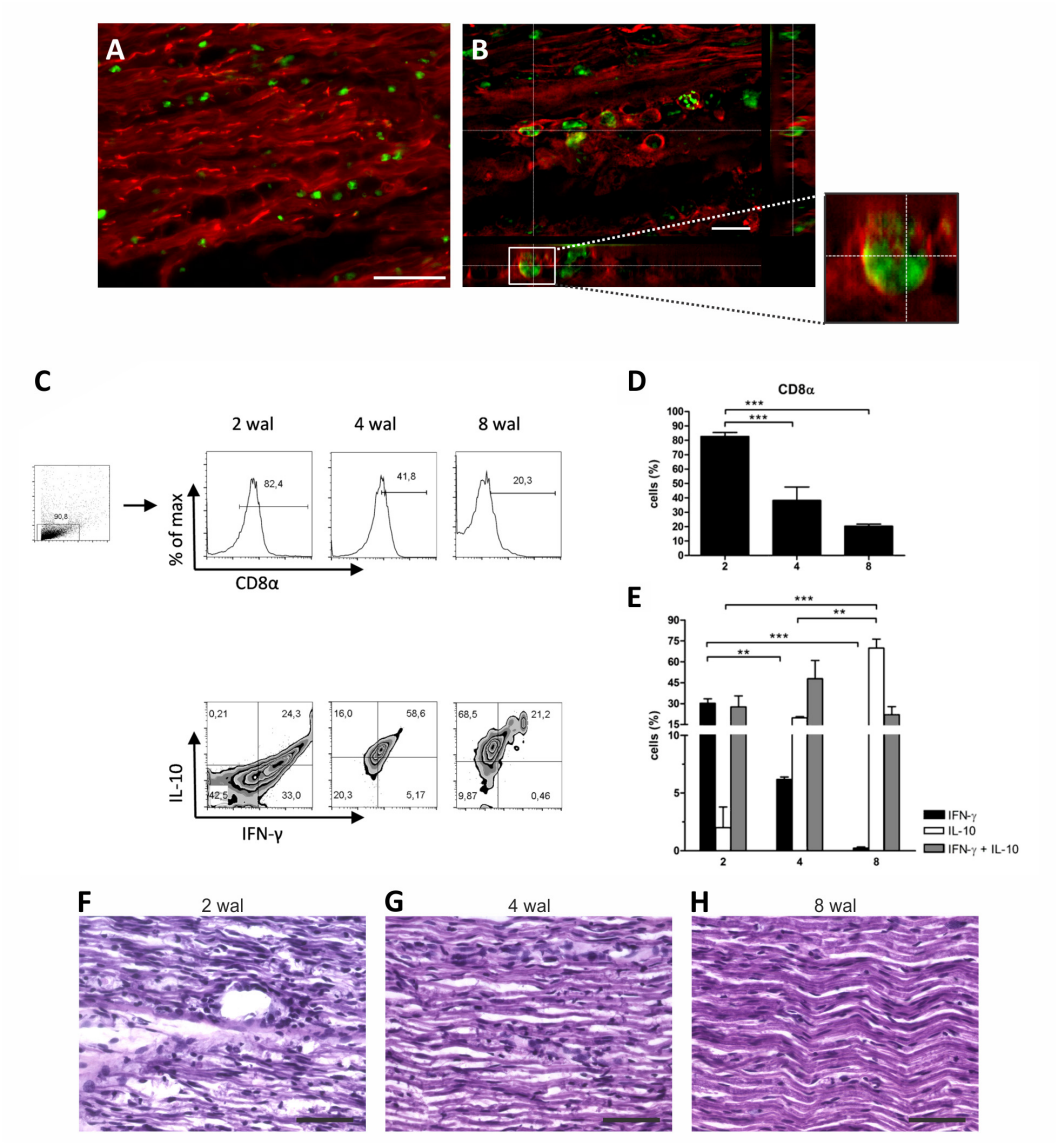
Crushed sciatic nerve CD8 T cell immunolabeling, quantification and phenotyping. A, B) Crushed sciatic nerve (2 weeks after lesion) was immunolabeled for CD8 T cells (green) and neurofilaments (axonal marker, red). Note CD8 T cells in the endoneural environment (B, detail). Scale bars: A, 50 μm; B, 20 μm. C-E) Crushed sciatic nerves were dissected at 2, 4 and 8 weeks after lesion (wal) and submitted to flow cytometry procedures. C) Representative graphs of CD8 T cell quantification (D) and cytokine expression (E). Note that the frequency of CD8 T cells diminishes over time (D), and these lymphocytes gradually express less INF-γ and more IL-10 (E). Data are presented as the mean ± SEM. n = 6 in each time point. **p<0.01; ***p<0.001 according to the one-way ANOVA followed by Bonferroni post-tests. F-H) H&E stained nerves at 2 (F), 4 (G) and 8 (H) weeks after lesion (wal), evidencing inflammation resolution. Scale bars: 50 μm.

Because CD8 T cells recognize cytosolic antigens via MHC-I and T cell receptor (TCR) interactions, we proceeded with CD8 T cell quantification and phenotyping in the crushed nerve. At 2 wal approximately 80% of the gated cells were CD8 T lymphocytes (Fig 3A–3D, S2 File), which then decreased to approximately 40% and 20% at 4 and 8 wal, respectively (Fig 3C and 3D). Moreover, the percentage of CD8 T cells producing the pro-inflammatory cytokine IFN-γ decreased over time, whereas the frequency of cells positive to the anti-inflammatory cytokine IL-10 increased during the regenerative process (Fig 3C and 3E, S2 File). It is worth to remember that according regeneration occurs, less inflammatory foci are seen in the tissue (Fig 3F–3H). As observed, the pattern of MHC-I expression and CD8 T cell infiltration were similar during the nerve regenerative process, suggesting MHC-I molecules might be involved in antigenic presentation rather than in axon growth control. Because PirB is a ligand of MHC-I molecules, we were interested in investigating whether it would present the same pattern of expression.

### Increased expression of PirB after sciatic nerve crushing

**Fig 4:**
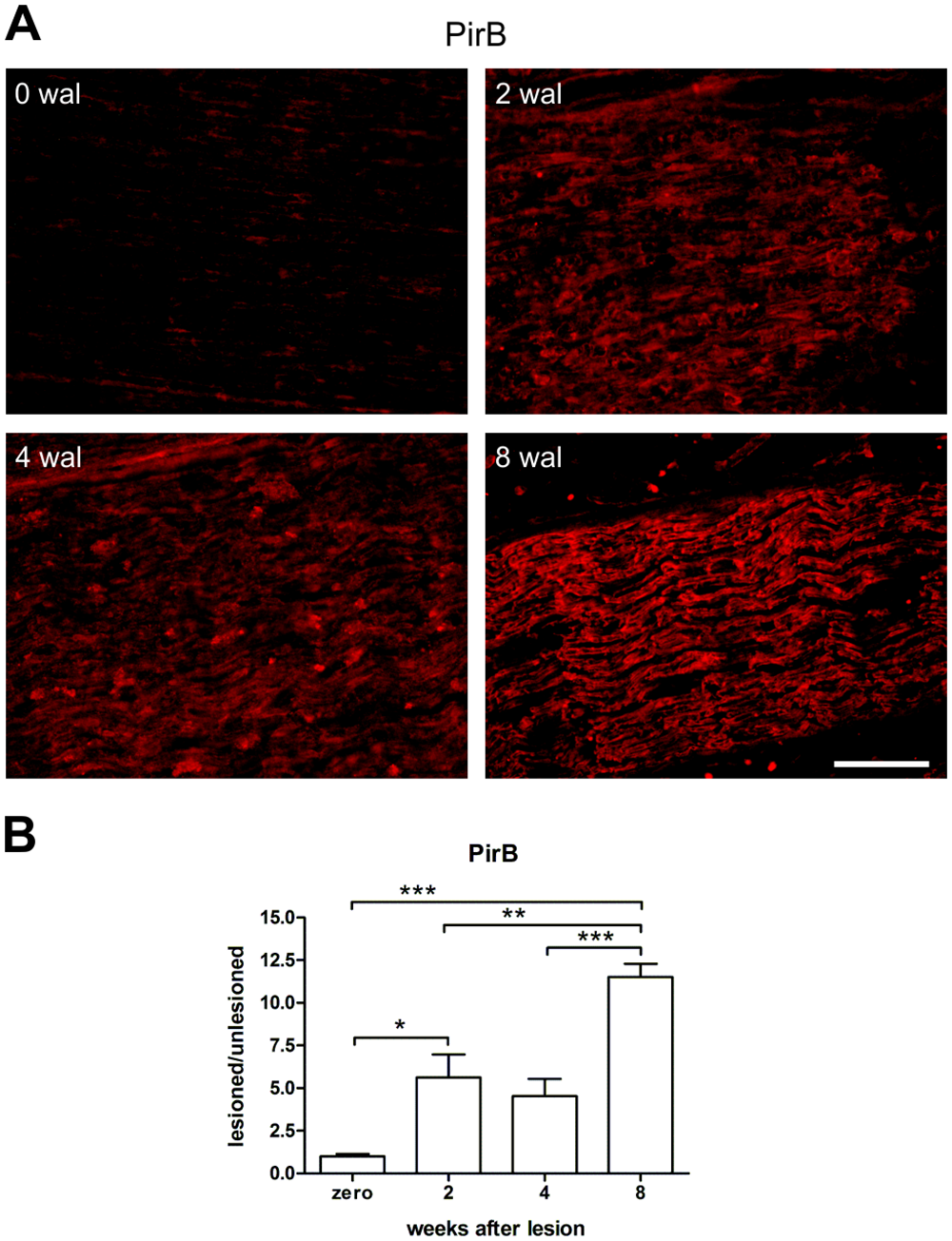
PirB expression in the crushed sciatic nerve. A) Representative images of PirB immunostained sciatic nerve at 2, 4, and 8 weeks after lesion (wal). Undamaged nerve was used as a control (zero wal). Scale bar: 100 μm. B) PirB expression quantification by the integrated density of pixels method, where the ratio lesioned/unlesioned was employed. Data are presented as the mean ± SEM. n = 6 in each time point. *p<0.05; **p<0.01; ***p<0.001 according to the one-way ANOVA, followed by Bonferroni post-tests.

**Fig 5:**
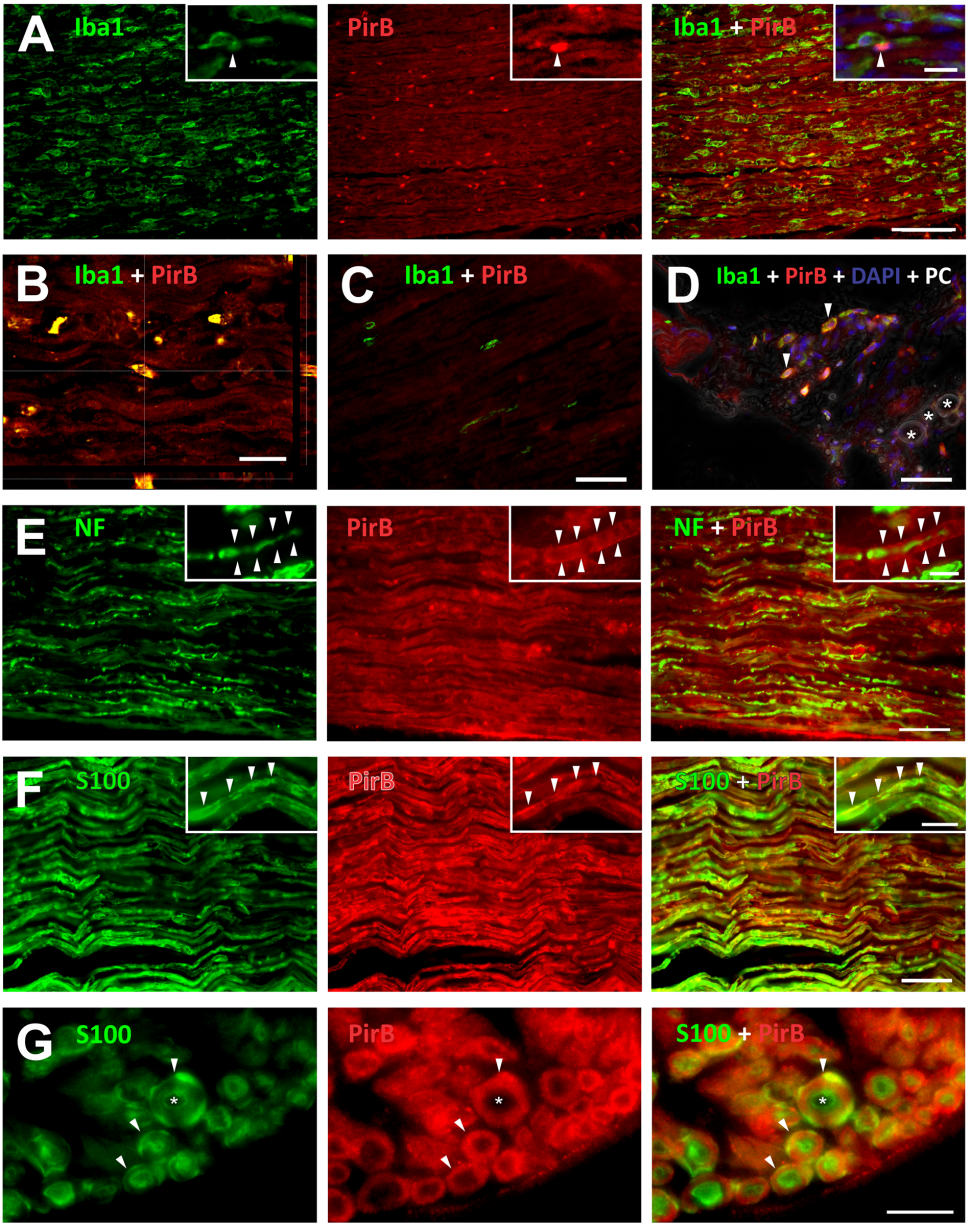
PirB expression in the sciatic nerve. Representative images of sciatic nerves double immunolabeled for PirB and A-D) Iba1 (macrophage marker), E) neurofilament (NF, axonal marker), or F and G) S100 (Schwann cell marker). A) PirB expression by macrophages was mostly restricted to round-shaped cells. Inset: macrophage expressing PirB (arrowhead); DAPI stained nuclei. B) Orthogonal projection of PirB^+^ macrophages. C) Macrophages from the undamaged nerve did not express PirB, whereas D) macrophages from the adjacent adipose tissue were PirB^+^ (arrowheads). Asterisks indicate adipocytes; DAPI stained nuclei, PC: phase contrast. E) Low or absent PirB expression by axons. In the detail an axon wrapped by PirB labeled Schwann cells (arrowheads). F) Schwann cells strongly expressing PirB (arrowheads in the detail). G) Transverse section of nerve fibers (arrowheads), showing PirB immunolabeling associated with the myelin sheath. Asterisk indicates the space occupied by the axon. Time-points: A and B, 2 weeks after lesion (wal); C and D, zero wal; E and G, 4 wal; F, 8 wal. Scale bars: A, 100 μm; B, 20 μm; C-F, 50 μm; G, 10 μm. Insets: A and F, 20 μm; B, 10 μm.

According to the immunohistochemistry analysis, PirB expression was very low under basal conditions but significantly increased in the damaged sciatic nerve at 2 wal, kept constant at 4 wal, and peaked at 8 wal (Fig 4A and 4B, S2 File). PirB was expressed on macrophages at all time-points post-injury. However, labeling was more intense on round-shaped macrophages resembling activated cells at 2 wal (Fig 5A and 5B). Curiously, fusiform macrophages presented low or no PirB immunolabeling, being few at 2 wal, and increasing thereafter. In the intact sciatic nerve, PirB^+^ macrophages were rarely observed (Fig 5C). Of note, in the adipose tissue adjacent to the non-operated nerve we found round-shaped macrophages that were highly PirB^+^ (Fig 5D). Although PirB was reported to be expressed by neuronal processes [18], we could not observe any evident overlapping of PirB and the axonal marker neurofilament at any time post-injury (Fig 5E) or under normal conditions. Regarding the Schwann cells, PirB was expressed by them on their cell bodies and extensions, both under basal conditions and all time-points evaluated after injury (Fig 5F). However, PirB immunolabeling was more prominent in such cells at 8 wal, a time point in which myelination was close to completion and when the Schwann cell S100 marker recovered its basal expression level (S3 Fig). It is remarkable that PirB was present on the surface of the Schwann cell membrane and associated with the myelin sheath (Fig 5G).

### Increased expression of MHC-I and PirB in the CNS following PNS damage

**Fig 6:**
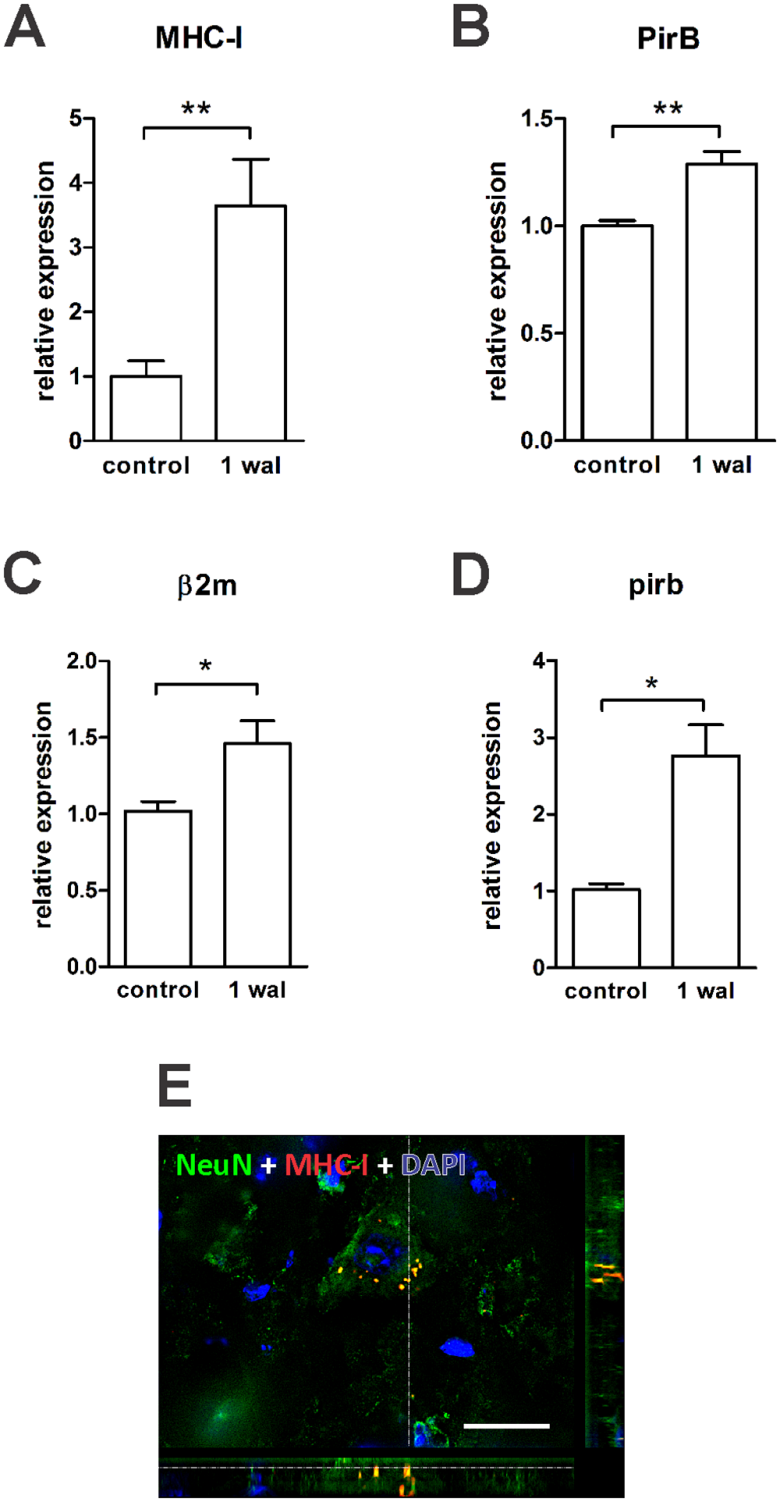
Increased protein and mRNA expression of MHC-I and PirB in the spinal cord following PNS damage. Mice were submitted to sciatic nerve transection. One week after lesion (wal), the spinal cord on the ipsilateral side was analyzed for MHC-I and PirB protein and gene expression. Uninjured mice were used as a control. MHC-I (A) and PirB (B) protein quantification by the integrated density of pixels method, applying the ratio lesioned/unlesioned. mRNA expression of the MHC-I β2 m chain (C) and PirB (D), relative to the control group. Data are presented as the mean ± SEM. n = 6 in each time point. *p<0.05; **p<0.01 according to the unpaired *t* test. E) Control neuron displaying cytoplasmic vesicles containing MHC-I molecules. Scale bar: 20 μm.

**Fig 7:**
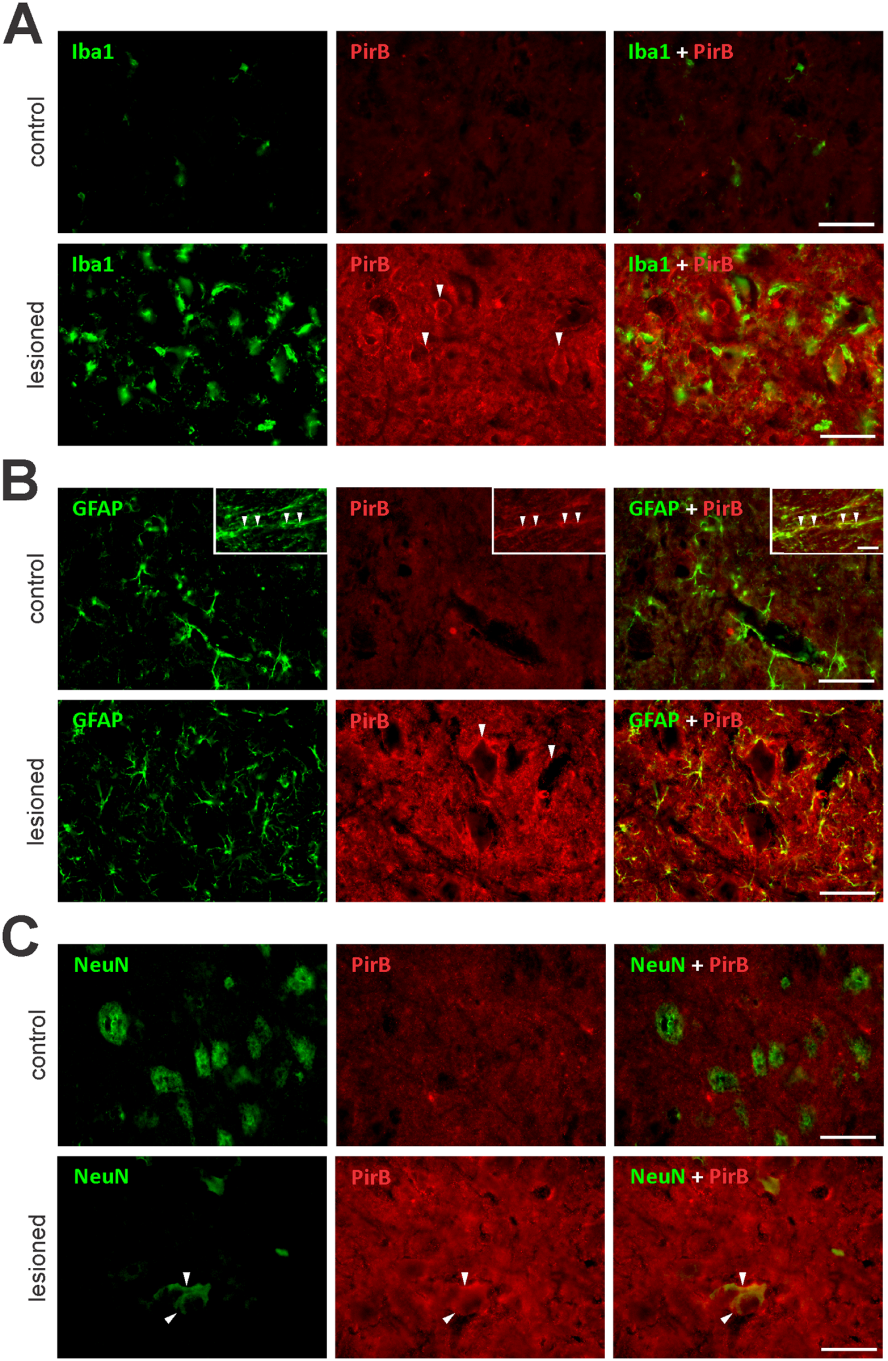
Spinal cord PirB immunolabeling is stronger in neuronal soma and processes. Following sciatic nerve transection, lumbar spinal cord sections were double-immunolabeled for PirB and A) Iba1 (microglia marker), B) GFAP (astrocyte marker), or C) NeuN (neuronal soma marker). Non-operated mice were used as a control. A) Low or null expression of PirB by microglia, either before or after nerve axotomy. B) Absent or low punctal expression of PirB by gray matter astrocytes. PirB immunolabeling could be visualized in white matter astrocytes (inset, arrowheads). C) PirB expression by neuronal soma surface and processes, under basal conditions and after injury. Arrowheads in A, B and C indicate neuronal soma expressing PirB. Scale bars: A-C, 50 μm; D, 20 μm.

We were interested in evaluating the spinal cord expression of MHC-I and its ligand (PirB) one week following sciatic nerve transection, when the injured limb is completely paralyzed. Sciatic nerve transection presents the advantage of producing CNS alterations, yet preserving the blood brain barrier at the spinal cord level [19]. Previously, our group demonstrated increased MHC-I expression in the spinal cord after peripheral nerve injury [6, 7] or during EAE [20], the labeling occurring in astrocytes, microglia, and neurons [6, 20]. Our current analysis also revealed increased protein and gene expression of MHC-I (Fig 6A and 6C, S2 File), as well as PirB (Fig 6B and 6D, S2 File) in the spinal cord following peripheral nerve transection. Interestingly, it was possible to observe MHC-I labeled cytoplasmic vesicles in neurons (Fig 6E) mainly in the undamaged group, in which the basal level of MHC-I protein was particularly low. PirB immunolabeling was weak on microglia (Fig 7A) and gray matter astrocytes (Fig 7B) before and after nerve transection. However, we could identify several fibrous astrocytes in the white matter displaying PirB (Fig 7B, inset). Regarding neurons, PirB labeling was visualized in the neuropil region under basal conditions and following injury (Fig 7C). Because PirB was reported to be expressed by peripheral macrophages [21], we were interested in investigating whether microglia could produce PirB *in vitro*. For this, we cultured microglia (S1 File) for 2, 4, 24, and 48 h and observed, surprisingly, PirB positive cells at all time-points (S4 Fig). Interestingly, PirB expression was lower on branched microglia than on round-shaped ones (S4 Fig).

## Discussion

An increasing number of studies have demonstrated the dual role that molecules classically considered as belonging to the immune system develop in the nervous system [22–25]. One example is the MHC-I molecule, widely known to present antigens to cytotoxic T lymphocytes (CTLs), which has been shown to be involved in CNS plasticity events [26, 27]. Accordingly, the importance of MHC-I for synapse refinement was first suggested by Huh and co-workers [4], who reported a surplus of retinogeniculate projections in mice lacking functional MHC-I molecules during development. Additionally, Oliveira *et al*.[8] proposed MHC-I to be involved in the maintenance of inhibitory synapses following PNS injury because fewer inhibitory terminals were found in contact with MHC-I-deficient spinal motoneurons. Thus, such inhibitory inputs are thought to be beneficial to the damaged neuron by shifting cell efforts towards regeneration instead of firing action potentials [8, 23]. Subsequent studies corroborated this hypothesis, connecting an increased expression of MHC-I to synapse detachment and maintenance of inhibitory inputs following axotomy [7, 9]. Although the role of MHC-I during CNS plasticity is not completely clear, evidence points to PirB as a key element for conveying this process. Primarily described as an immune system molecule involved in cell silencing [28], PirB was visualized on axons and dendrites of different subsets of neurons [14, 18, 29], as well as in the axon growth cone close to or at the vicinity of the synapse [14]. Syken and colleagues demonstrated that PirB binds to neurons in an MHC-I-dependent manner and possibly stabilizes neuronal circuits because a robust ocular dominance in the visual cortex was also observed in mice lacking functional PirB [14]. Herein, we demonstrate that PirB and MHC-I expression are enhanced in spinal cord neurons following PNS injury, suggesting a possible participation of such molecules during neuronal plasticity events. On the contrary, in the crushed nerve we observed no axon labeling of PirB at all time-points studied, and only a few MHC-I labeled axons were depicted. Of note, in the crushed nerve, MHC-I protein expression and CD8 T lymphocytes infiltration displayed similar patterns, peaking at two weeks after lesion and decreasing thereafter. Additionally, at two weeks after crushing, most of the MHC-I immunolabeling was restricted to glial and infiltrating cells, especially to macrophages. Considering that CD8 T cells are involved in antigen recognition via TCR and MHC-I interaction, together with the kinetics of MHC-I expression, it seems reasonable that in the nerve, MHC-I greatly contributes to the inflammatory immune response following injury rather than the axonal plasticity.

The involvement of PirB on controlling events of cellular plasticity and motility has been the subject of many studies [11, 12, 26, 30, 31]. In this way, Pereira and colleagues [11] showed that bone marrow derived macrophages lacking PirB are hyperadhesive and spread more rapidly *in vitro* than WT cells, as the result of phosphorylation and activation of integrin signaling pathway proteins. Moreover, it has also been demonstrated that polymorphonuclear neutrophils are hyperadhesive in the absence of PirB, and they present an enhanced respiratory burst and secondary granule release mediated by integrin signaling [11]. Although PirB was reported to be expressed by resting macrophages [21], in the present study we did not observe strong PirB immunolabeling on microglia and nerve resident macrophages from non-operated animals, which displayed a classic branched phenotype [32, 33]. We hypothesize they are possibly involved with tissue integrity surveillance. Considering the inhibitory effects of PirB on cellular plasticity, together with the fact that microglial cells are very motile under normal conditions [32, 34], it seems reasonable that resting microglia, as well as macrophages from unlesioned nerves, do not express PirB, so they can continuously move throughout the nervous system microenvironment. This hypothesis is in line with our *in vitro* evaluation, in which round-shaped microglia adhered to a PLL-treated plate strongly expressed PirB. Hypothetically, the lack of PirB expression by those cells would benefit prompt activation under slight stimuli. Under allostatic conditions, the expression of PirB by macrophages correlates with cell inactivation mechanisms, as demonstrated during experimental colitis where PirB^-/-^ macrophages increased the production of pro-inflammatory cytokines (IL-6, IL-1β, and TNF-α), as well as kinases involved in their synthesis [35]. Furthermore, in an experimental model of idiopathic pulmonary fibrosis, alveolar macrophages lacking PirB displayed increased expression of profibrinogenic markers and aggravated the disease [36]. This correlates with the low level of PirB immunolabeling by spinal cord microglia following nerve transection, reflecting the activation state presented by those cells that continued to remain active one week after [37]. Regarding PNS PirB expression by nerve macrophages, we found that it was stronger two weeks after crushing, a period in which most myelin debris had already been removed and macrophages were undergoing inactivation [38, 39]. Thereafter, as regeneration took place, it was expected that macrophages would downregulate PirB expression and recover the basal phenotype. Of note, MHC-I molecules were more prominent on nerve macrophages two weeks after lesion. Although it is possible that MHC-I may be interacting with PirB in a cis or trans fashion on nerve macrophage cell membranes, this does not greatly contribute to the inactivation of those phagocytes because the anti-inflammatory immune response is naturally developed for that purpose [38–40]. Thus, the MHC-I expression by these cells seems to be more closely related to the antigen-presenting process, as previously discussed.

Gray matter astrocytes eventually displayed PirB immunolabeling after PNS injury, mostly on fine cell projections. The transection of the sciatic nerve is known to cause increased astrogliosis accompanied by a detachment of synaptic inputs in apposition to the damaged neuron [7, 9, 41]. Considering that synaptic stripping requires astrocyte motility, one could expect that astrocytes would not express high levels of PirB. In agreement with this, astrocytes from the white matter without contact to the perikarya were PirB positive. Of note, Deng *et al*. [18] reported PirB expression by CNS astrocytes following intrahippocampal LPS injection. However, it occurred one month after the endotoxin-induced neuroinflammation.

In addition to binding to MHC-I molecules, PirB was reported to be a functional receptor for the myelin axonal growth inhibitory proteins Nogo66, MAG, and OMgp, which blocked neuronal outgrowth [12]. Moreover, it has been demonstrated that PirB interacts with neurotrophin receptors, which may in turn also impair neurite elongation [31, 42]. Although the role of PirB in the PNS remains poorly understood, Thams *et al*. [15] showed its expression by a subpopulation of Schwann cells in mice, suggesting an interaction with MHC-I-expressing axons, possibly in an inhibitory fashion. Herein, we show enhanced PirB expression in the sciatic nerve following crush injury and peaking at eight weeks after lesion, a period of clearly decreased expression of MHC-I molecules. Of note, PirB immunolabeling was more prominent in the Schwann cells, also being associated with the myelin sheath. In view of the opposing patterns of PirB and MHC-I expression in the crushed nerve, our data implies that other molecules besides MHC-I may be the main ligand of PirB in Schwann cells during the nerve regenerative process. In view of the role of PirB in cellular plasticity and motility, we suggest that the interaction of PirB and MAG, for example, may trigger inhibitory signals, and help control myelination. Regarding the expression of MHC-I by few axons, it is also possible that sprout growth inhibition is mediated by Schwann cells via PirB [15].

In summary, our data reinforce the concept that MHC-I and PirB interact in the CNS during the neuronal plasticity process, whereas in the PNS, such molecules seem to work differently to what is reported in the CNS. In this way, we believe that in the damaged nerve MHC-I acts as an antigen presenting to CTLs whereas PirB inhibits macrophage and glial activation at the final stages of Wallerian degeneration. Moreover, our results shed light to an unforeseen PirB mechanism that ensures cell motility to resident PNS macrophages under normal conditions and Schwann cells during axonal degeneration and regeneration.

## Supporting Information

S1 Fig Schematic representation of image acquisition fields for immunolabeling quantification. A) Longitudinal section of the sciatic nerve, immediately distal to the crush site, where “x” represents fields of interest. P and D, proximal and distal to the lesion site, respectively. B) Transversal section of the lumbar intumescenses, showing the acquisition field (dashed line) in the ventral horn. IL, ipsilateral; CL, counter-lateral.
(TIF)

S2 Fig Motor function evaluation. Mice were submitted to the automated walking track test before (-1 dal) and after (3–56 dal) the left sciatic nerve crushing and the sciatic function index (SFI) was calculated (arbitrary units). Data are presented as mean ± SEM. n = 18 (-1–14 dal), n = 12 (16–28 dal), n = 6 (31–56 dal).
(TIF)

S3 Fig Schwann cells marker basal level is recovered eight weeks after sciatic nerve crushing. A) Representative images of the Schwann cell marker S100 at 2, 4 and 8 weeks after lesion (wal). Undamaged nerve was used as control (zero wal). Scale bar: 100 μm. B) Quantification of S100 proteins by the integrated density of pixels method, applying the ratio lesioned/unlesioned. Data are presented as mean ± SEM. n = 6 in each time point. **p<0.01; ***p<0.001 according to the one-way ANOVA, followed by Bonferroni post-tests.
(TIF)

S4 Fig PirB expression by adherent microglia *in vitro*. Isolated primary culture derived-microglia were seeded in 24-well plates for 2, 4, 24 or 48h, fixed and immunostained for Iba1 (microglia marker) and PirB. Microglia expressed PirB in all of the time-points analyzed. However, PirB expression is generally low in cell branches (arrowhead) and high in cell bodies (arrow). Scale bar: 50 μm.
(TIF)

S1 File Methodology. Functional analysis and Microglia cell culture protocols.
(DOCX)

S2 File Raw data. Immunolabeling, flow cytometry and real time PCR quantification.
(PZF)
