# Correction: MHC-I and PirB Upregulation in the Central and Peripheral Nervous System Following Sciatic Nerve Injury

**DOI:** 10.1371/journal.pone.0165185

**Published:** 2016-10-17

**Authors:** André Luis Bombeiro, Rodolfo Thomé, Sérgio Luiz Oliveira Nunes, Bárbara Monteiro Moreira, Liana Verinaud, Alexandre Leite Rodrigues de Oliveira

There is grant information missing from the Funding Disclosure. The statement should read: This work was supported by the São Paulo Research Foundation (FAPESP). Grants were provided by the following Brazilian agencies: FAPESP (2011/08712-4, 2012/20456-6; 2014/06892-3), CAPES and CNPq (300553/2013-9).
