# Supplementary figures and images for: MHC-I and PirB Upregulation in the Central and Peripheral Nervous System following Sciatic Nerve Injury
Source: PLoS One. 2016 Aug 23;11(8):e0161463. doi: 10.1371/journal.pone.0161463 (PMC4995013; doi:10.1371/journal.pone.0161463)

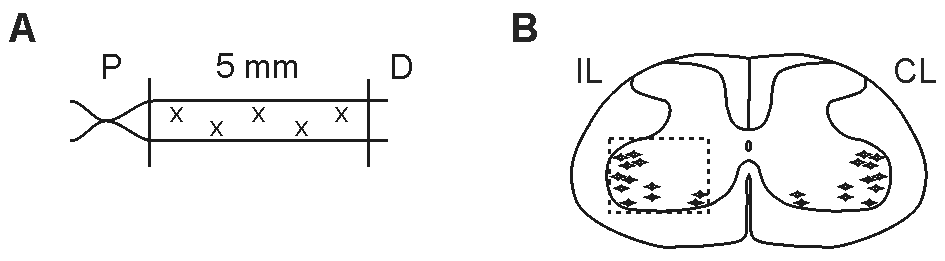

Supplement: S1 Fig — A) Longitudinal section of the sciatic nerve, immediately distal to the crush site, where “x” represents fields of interest. P and D, proximal and distal to the lesion site, respectively. B) Transversal section of the lumbar intumescenses, showing the acquisition field (dashed line) in the ventral horn. IL, ipsilateral; CL, counter-lateral. (TIF) [file pone.0161463.s001.tif]

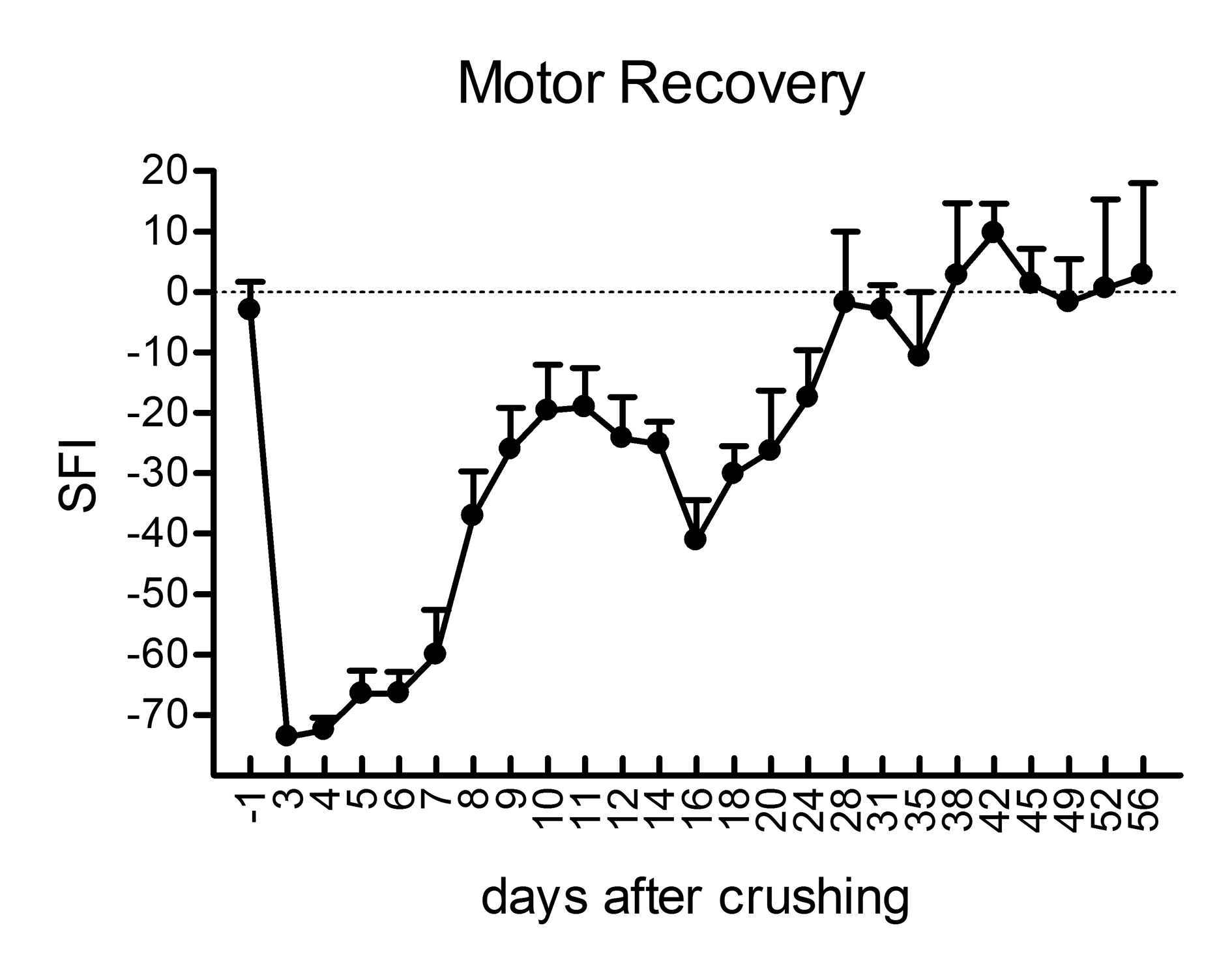

Supplement: S2 Fig — Mice were submitted to the automated walking track test before (-1 dal) and after (3–56 dal) the left sciatic nerve crushing and the sciatic function index (SFI) was calculated (arbitrary units). Data are presented as mean ± SEM. n = 18 (-1–14 dal), n = 12 (16–28 dal), n = 6 (31–56 dal). (TIF) [file pone.0161463.s002.tif]

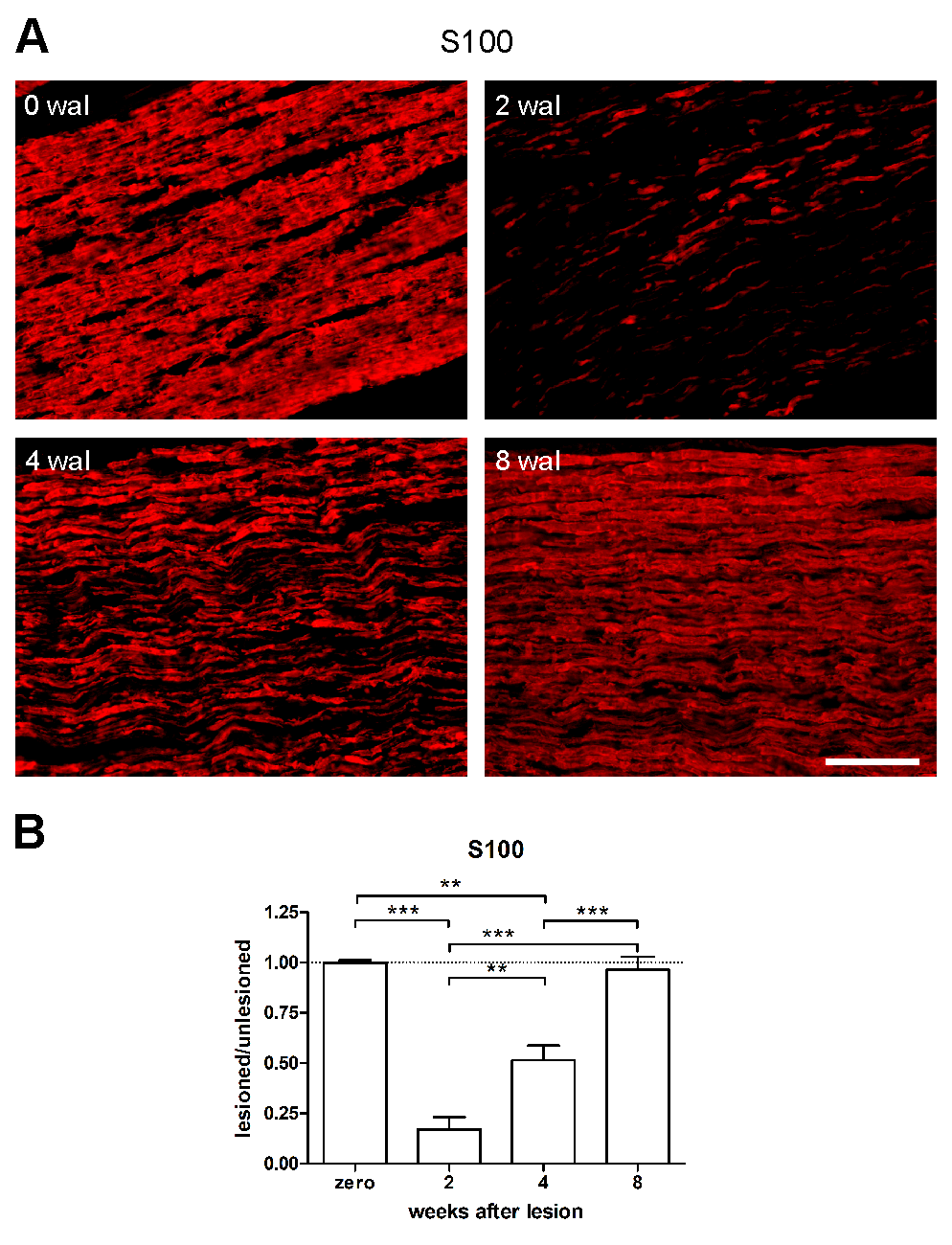

Supplement: S3 Fig — A) Representative images of the Schwann cell marker S100 at 2, 4 and 8 weeks after lesion (wal). Undamaged nerve was used as control (zero wal). Scale bar: 100 μm. B) Quantification of S100 proteins by the integrated density of pixels method, applying the ratio lesioned/unlesioned. Data are presented as mean ± SEM. n = 6 in each time point. **p<0.01; ***p<0.001 according to the one-way ANOVA, followed by Bonferroni post-tests. (TIF) [file pone.0161463.s003.tif]

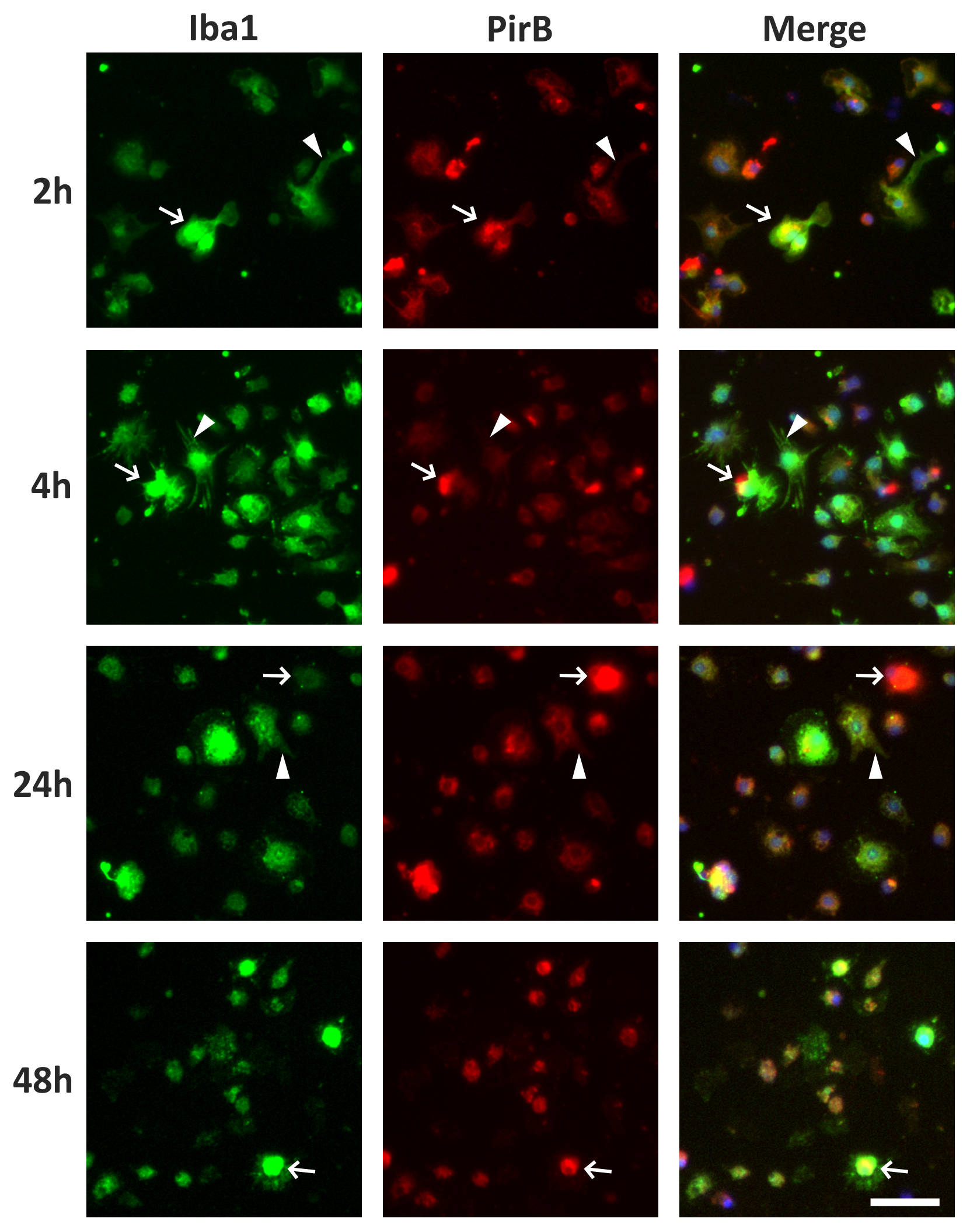

Supplement: S4 Fig — Isolated primary culture derived-microglia were seeded in 24-well plates for 2, 4, 24 or 48h, fixed and immunostained for Iba1 (microglia marker) and PirB. Microglia expressed PirB in all of the time-points analyzed. However, PirB expression is generally low in cell branches (arrowhead) and high in cell bodies (arrow). Scale bar: 50 μm. (TIF) [file pone.0161463.s004.tif]
