# Supplementary material for: MHC-I and PirB Upregulation in the Central and Peripheral Nervous System following Sciatic Nerve Injury
Source: PLoS One. 2016 Aug 23;11(8):e0161463. doi: 10.1371/journal.pone.0161463 (PMC4995013; doi:10.1371/journal.pone.0161463)
Supplement: S1 File — Functional analysis and Microglia cell culture protocols. (DOCX) [file pone.0161463.s005.docx]

**Supporting Information**

**MHC-I and PirB Upregulation in the Central and Peripheral Nervous System Following Sciatic Nerve Injury**

**Authors**: André Luis Bombeiro, Rodolfo Thomé, Sergio Luiz Nunes Oliveira, Bárbara Monteiro Moreira, Liana Verinaud and Alexandre Leite Rodrigues de Oliveira.

**Functional analysis**

The motor function was evaluated by the Sciatic Function Index (SFI), using the automated Cat Walk System (Noldus Inc., The Netherlands; <http://www.noldus.com/animal-behavior-research/products/catwalk>) as previously described [[1](#_ENREF_1)]. In this system, animal is allowed to walk on a glass platform which is illuminated with a green light, enhancing the footprints when paws contact the surface. The run is recorded by a high speed camera under the walkway and data are transferred to a host computer, where they are analyzed by software (CatWalk XT 10.5). The SFI was calculated according to the following formula [[2](#_ENREF_2)]: SFI=118.9((ETS-NTS)/NTS)-51.2((EPL-NPL)/NPL)-7.5, where N, normal or non-operated side; E, experimental or crushed side; PL, print length and TS, toe spread. For adaptation purpose, animals were placed in the system to walk 5 minutes per day, during three days, before experiments begin.

**Microglia cell culture**

Primary glial cell culture and microglia isolation were carried out as previously described [[3](#_ENREF_3)]. In brief, 3 to 5 days-old newborn C57BL/6 mice (n=10, obtained from CEMIB/UNICAMP) were deeply anesthetized on ice and decapitated. The brain of each pup was dissected out on ice-cooled PBS followed by meninges removal from the cerebral hemispheres, which were dissociated by mechanical trituration and cultured in medium DMEM F12 (Gibco, USA) containing 10% FCS (Invitrogen, Germany) and 1% penicillin/streptomycin (Nutricell, Brazil) during 14 days (37°C, 5% CO_2_). Flasks were shacked (200rpm, 2h) and the microglia enriched supernatant was collected. Cells were harvested in a 24 well plate (2x10^5^ cells/well) and washed with PBS 2h latter to remove the non-adherent cells. 2, 4, 24 and 48h after seeding, cultures were fixed with ice-cooled paraformaldehyde 4% (30min) for the immunofluorescence procedures. Experiments were done in triplicate. Purity of isolated microglia was higher than 95%, as confirmed by flow cytometry employing CD11b FITC-conjugated antibodies (BD Biosciences, Cat. code: 553310).

**References**

1. Barbizan R, Castro MV, Rodrigues AC, Barraviera B, Ferreira RS, Oliveira AL. Motor recovery and synaptic preservation after ventral root avulsion and repair with a fibrin sealant derived from snake venom. PLoS ONE. 2013;8(5):e63260. Epub 2013/05/15. doi: 10.1371/journal.pone.0063260. PubMed PMID: 23667596; PubMed Central PMCID: PMC3646764.

2. de Medinaceli L, Freed WJ, Wyatt RJ. An index of the functional condition of rat sciatic nerve based on measurements made from walking tracks. Experimental neurology. 1982;77(3):634-43. PubMed PMID: 7117467.

3. Bombeiro AL, Goncalves LA, Penha-Goncalves C, Marinho CR, D'Imperio Lima MR, Chadi G, et al. IL-12p40 Deficiency Leads to Uncontrolled Trypanosoma cruzi Dissemination in the Spinal Cord Resulting in Neuronal Death and Motor Dysfunction. PLoS ONE. 2012;7(11):e49022. PubMed PMID: 23152844.
